# An anomaly detection method for identifying locations with abnormal behavior of temperature in school buildings

**DOI:** 10.1038/s41598-023-49903-7

**Published:** 2023-12-21

**Authors:** Ashani Wickramasinghe, Saman Muthukumarana, Matt Schaubroeck, Surajith N. Wanasundara

**Affiliations:** 1 Department of Statistics, Faculty of Science, University of Manitoba, Winnipeg, R3T 2N2 Canada; 2 ioAirFlow, Winnipeg, R3C 3Z5 Canada

**Keywords:** Environmental impact, Information technology, Statistics

## Abstract

Time series data collected using wireless sensors, such as temperature and humidity, can provide insight into a building’s heating, ventilation, and air conditioning (HVAC) system. Anomalies of these sensor measurements can be used to identify locations of a building that are poorly designed or maintained. Resolving the anomalies present in these locations can improve the thermal comfort of occupants, as well as improve air quality and energy efficiency levels in that space. In this study, we developed a scoring method to identify sensors that shows collective anomalies due to environmental issues. This leads to identifying problematic locations within commercial and institutional buildings. The Dynamic Time Warping (DTW) based anomaly detection method was applied to identify collective anomalies. Then, a score for each sensor was obtained by taking the weighted sum of the number of anomalies, vertical distance to an anomaly point, and dynamic time-warping distance. The weights were optimized using a well-defined simulation study and applying the grid search algorithm. Finally, using a synthetic data set and the results of a case study we could evaluate the performance of our developed scoring method. In conclusion, this newly developed scoring method successfully detects collective anomalies even with data collected over one week, compared to the machine learning models which need more data to train themselves.

## Introduction

Anomaly detection is one of the most popular research areas in time series data mining. A data point that does not follow the pattern of the rest of the data can be considered an anomaly or outlier. Identifying these anomaly points is important for many industries. Some applications include the detection of abnormal behavior of ECG signals in the health industry^1^, credit card fraud detection in the banking industry^2^, anomalous behavior in aircraft^3^, identifying spammers, online fraudsters in social media^4^ and many more^5^.

Nowadays commercial buildings also provide an opportunity to monitor indoor environment quality with the help of Internet of Things (IoT) sensors. These devices measure the environment of the building and generate temporal data which helps building owners to understand the indoor environmental quality (IEQ) and thermal comfort of the tenants. In another research work, we used these sensor data to identify locations with similar thermal environments of a building^6^. Identifying the issues of the indoor thermal environment is important for building owners to save energy and keep tenants comfortable. In order to identify these issues, we can inspect IoT sensor data and detect abnormal behaviors. The presence of these abnormal behaviors might be occurred due to the issues of the location of those sensors are located.

Existing anomaly detection algorithms can be mainly divided into two categories: supervised and unsupervised. Supervised anomaly detection in sensor data refers to the process of identifying abnormal events or patterns in data using labeled training data. The supervised anomaly detection methods have certain drawbacks including limited anomaly labeling, sensitivity to labeling errors, difficulty to identify novel anomalies and insufficient data. Due to these limitations, most anomaly detection methods use unsupervised or semi-supervised techniques. In 2021, Yan^7^ introduced a generative adversarial network-based (GAN^8^) chiller fault detection framework. However, the framework used a labeled training dataset, which identifies the specific fault to generate synthetic data. In our study, where we work with Internet of Things (IoT) sensor data, we do not have any labels, and the anomaly patterns are subject to change. Because of these reasons, unsupervised methods are more suitable for our study.

Unsupervised methods in anomaly detection do not rely on labeled anomaly data during training. Instead, they learn patterns and structures inherent in the data to detect anomalies based on deviations from the learned normal behavior. Some state-of-the-art approaches for unsupervised anomaly detection include Robust KDE (RKDE)^9^, Local Outlier Factor (LOF)^10^, mixture models (EGMM)^11^, one-class SVM (OCSVM)^12^, and autoencoders^13^, Isolation Forest (IForest)^14^. However, unsupervised methods also may have limitations in certain cases, such as difficulty in identifying the types of anomalies or sensitivity to the data distribution. Most of the unsupervised methods rely on the distribution and need to have proper parameter tuning. Quintana and the team have used Automated Load profile Discord Identification(ALDI)^15^ to identify outliers in energy load profiles. But this method identifies outliers by testing a hypothesis for significantly different time series distributions. In our method we do not rely on the distribution, but the temporal behavior of the time series.

Transitioning to the application of this framework in building science, previous studies have primarily concentrated on pinpointing abnormal data points in a time series. However, a single abnormal point may not necessarily signify a problem within a specific building location. For a more comprehensive understanding, we must look beyond single-sensor anomalies and instead concentrate on clusters of abnormal behaviors across all sensors on a building floor. When the same abnormal behavior at the same time is observed in multiple sensors, it suggests a higher likelihood of a building anomaly. This approach allows us to identify potential issues with the building’s HVAC system, control system, external envelope, or other infrastructure problems.

In 2019, Wang and his team has proposed an anomaly detection framework for thermal comfort in buildings^16^. It is a stochastic-based, two-step anomaly detection framework that is based on occupants’ votes. An outlier is automatically flagged when a vote is significantly different from other occupants’ votes. In the building industry, Fault diagnosis and detection (FDD) is an application of anomaly detection that monitors building HVAC systems to identify faults^17^. Graph-based anomaly detection methods also has been introduced by some researchers^18,19^ Another research work^20^ was done to detect anomalies in indoor office space by predicting values using long short-term memory (LSTM). They have used IoT sensors to collect temperature and humidity data.

The majority of existing research^21–24^ have considered identifying abnormal points of a single time series (intra-time series anomaly detection). But, these single abnormal points can be raised due to equipment failures within the data collection hardware itself, which may lead to a false positive or false negative. Lieu et al.^21^ have used energy data, and a data point, which has fallen outside the $95\%$ confidence bounds, is considered as an abnormal consumption. By dividing univariate time series into subsequences, Debanjana and Harry^22^ could classify a data point as an anomaly or normal point using one-class support vector machines (OC-SVM) in 2020. In Wei et al.’s paper^23^, an unsupervised temperature anomaly detection method is proposed to detect anomalies in real-time temperature time series. It sets dynamic thresholds based on the Smoothed Z-Score Algorithm.

The importance of our study is that it identifies abnormal time series compared to every time series that we use in a study (inter-time series anomalies) and proposes a novel method to identify collective anomalies which can occur due to environmental issues. Furthermore, this method operates without the need for a labeled large training dataset and does not depend on the distribution of time series data. Ultimately the locations of these sensors can be considered as the locations that indicate a potential issue within the HVAC system or building construction. The scoring method development considered the number of anomalies, the vertical distance between the average point and an abnormal point, and the DTW distance between the average time series and the given time series. In the end, we applied the developed scoring method in a school building, located in New York, USA, and a synthetic data set, and discussed the results.

## Methods

### Anomaly detection

**Figure 1:**
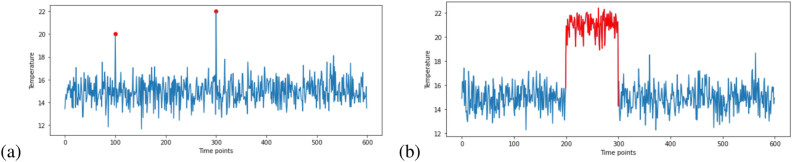
(**a**) Contextual anomalies: due to hardware issue, (**b**) collective anomalies: due to environmental issue.

An anomaly is something that deviates from what is standard or expected^25^. The anomaly detection problem for time series is usually formulated in a way that can identify outlier data points relative to some usual signal. Anomaly detection within the context of buildings has real-world implications. Anomalies in building IEQ data can be caused by either environmental issues or hardware issues. A collective anomaly could identify inefficient controls or HVAC systems or could point to occupant behaviors negatively impacting the energy use within that space^26^. Contextual anomalies (as defined by a greater variation from the average within that same space) could identify failing or poorly calibrated hardware, or failure points within a building’s mechanical or construction environment. Figure 1 shows the difference between these contextual and collective anomalies.

There are many approaches to detecting an anomaly in time-series data^27^ and a few common methods are presented in Table 1. Seasonal Trend Decomposition^28^ is one of the classic methods, and it uses a threshold to identify anomalies. Isolation Forest^29^ is based on the classification models, and it does not consider any distance measures. The forecasting method^30^ predicts the next point and based on the identified pattern from the past data it tries to identify anomalies. All these three methods have different methods of identifying anomalies. Hence we applied these three anomaly detection methods and DTW based anomaly detection method, which is explained in the next section, to sample time series data and selected the best method. The best method should identify collective anomalies, as they represent environmental issues.

**Table 1:** Anomaly detection methods, used in this study to compare with the DTW-based method.

Method	Summary
Seasonal trend decomposition^28^	It only uses residue data from the decomposition to identify anomalies. This algorithm calculates the deviation of residue and uses a threshold to identify anomalies
Isolation Forest method^29^	It randomly selects a feature from the given set of features and then randomly selects a split value between the max and min values of that feature to isolate the outliers
Forecasting method^30^	The forecasting method is based on the approach that generates a predicted value of the next point, by considering several points from the past

#### Anomaly detection method based on dynamic time warping

This method is based on the anomaly detection method, which was introduced by Diab and team^31^. It has a control time series and a data time series. Anomalies are identified based on the distance between points of the optimal path. The optimal path means the optimal match between the control series and the given time series. The basic steps of this algorithm are as follows:

1. Identify the optimal similarity path between two series (using DTW).
2. Calculate Euclidean distances between those points in the optimal path.
3. Calculate median absolute deviation (MAD) of distances.
4. Consider the points which are 3 times MAD away from the median as outliers.
5. Three or more consecutive outliers are considered as anomalies.

The importance of this algorithm is, it considers three or more consecutive outliers as anomalies. It detects only the collective anomalies, which are caused by environmental issues. However, this algorithm identifies anomalies based on a control sequence, but in practical situations, we will not always be able to find control sequences. Also, this method detects anomalies within single time series. But in our study, we want to identify abnormal time series compared to the all time series (inter-time series anomaly detection). Then it helps to identify abnormal sensors and locations with issues. Due to these limitations, we did some alterations to the existing method in order to apply it in our research study.

Instead of the control sequence, we used the median time series, which was created by calculating the median values of all the time series at each time point. Also when calculating the MAD value, we calculated all the distance values, comparing the median time series with each time series. By calculating MAD in that way, we could consider all the time series and identify the anomalies.

#### Dynamic time warping

Dynamic Time Warping (DTW)^32^ measures the distance between two arrays or time series. DTW is a method that calculates an optimal match between two given sequences. This method allows us to find the distance between sequences of different lengths. Let *A* and *B* be two sequences with length $L_A$ and $L_B$ respectively. $a_i$ and $b_j$ indicate the *i*th and *j*th observations of *A* and *B* respectively. Then the pairwise euclidean distances^33^ can be calculated for each observation of *A* and *B*. It will yield the $L_A \times L_B$ distance matrix *S*. The cumulative distance matrix *D* is calculated as in Eq. (1). The matrix *D* captures the total cost of alignment between the $a_1,b_1$ and $a_{L_A},b_{L_B}$. A lower total cost shows a higher similarity between the two sequences.

1 $$\begin{aligned} D_{i,j} = min(D_{i-1,j},D_{i,j-1},D_{i-1,j-1}) + S_{ij} \end{aligned}$$

In the above equation $i=1,\ldots ,L_A$, $j=1,\ldots ,L_B$ and $S_{ij} = d(a_i,b_j)$. The final distance between *A* and *B* is the bottom corner value of the cumulative distance matrix *D*, which is $D(L_{A},L_B)$

#### Median absolute deviation

The Median Absolute Deviation (MAD) is a robust measure of how a set of data is spread out. The variance and standard deviation are also measures of spread, but they are more affected by extremely high or extremely low values. Also, in practical situations, it is hard to get normally distributed data. Hence, the MAD is one statistic that we can use instead. It is less affected by outliers because outliers have a smaller effect on the median than they do on the mean. MAD is defined as follows,

2 $$\begin{aligned} MAD = median(|X_i - median(X)|) \end{aligned}$$

Here $X_i$ represents *i*th observation and according to our study *X* means the list distances between each data point of the optimal path, created using each time series and the control time series.

### Abnormal sensor detection method

In this section, we explain the method we developed to identify sensors that can be considered abnormal compared to a group of sensors positioned within a building. The locations where those abnormal sensors are located can be considered as problematic areas of the building. Algorithm 1 summarizes the process of our algorithm.

**Algorithm 1:**
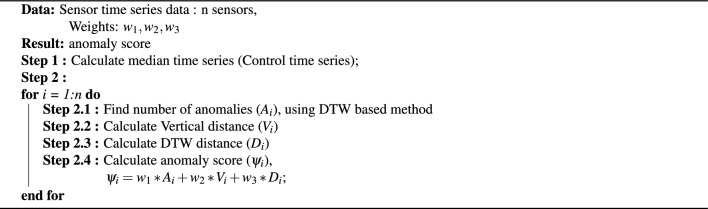
Abnormal sensor detection.

We developed a scoring method to identify abnormal sensors, and identifying the main parameters which help to detect differences between two-time series was important. Considering literature and observations we decided to use the following parameters to compare the difference between the control and the given time series:

1. Number of anomalies. (based on section “Anomaly detection method based on dynamic time warping”).
2. Median of vertical Euclidean distances between outlier points, and control time series.
3. DTW distance between time series (check the similarity of the patterns).

We calculated the above values for all the sensors and normalized using min-max normalization and bring all values into one scale, from 0 to 1. Not all these three parameters have the same level of importance when detecting the difference between control and other time series. Hence, we considered generating a weighted score as above in algorithm 1, step 2.4, and ranking the time series based on these scores. However, since there is no historical data, on which are labeled as abnormal or not, optimizing the weights in a way that the abnormal sensors get the highest score was another challenge. Hence to overcome this, we had to develop a method to scale the weights using a simulation study.

### Simulation study

**Figure 2:**
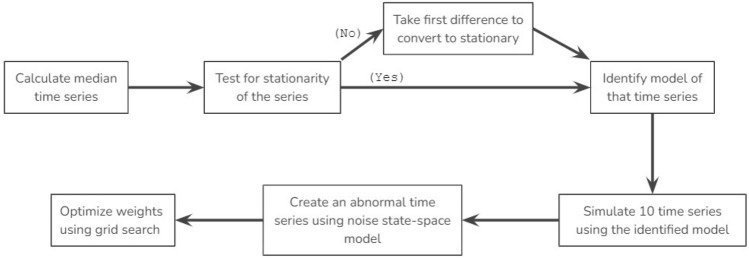
Flow chart of the simulation study.

Since we have no information about the actual abnormal sensors, we developed a simulation study to generate a set of ‘normal’ time series, and an ‘abnormal’ by considering the control time series. The flow chart in Fig. 2 summarizes the simulation work.

First, we had to identify the time series model of the median time series in order to generate a set of normal time series. Here we considered auto-regressive model (AR), moving-average model (MA), Auto Regressive Moving Average (ARMA), and Auto-Regressive Integrated Moving Average (ARIMA) models which are expressed using the Eqs. (3), (4), (5) and (6) respectively. In these equations, $y_t$ means *y*, response variable, measured at time *t*, and $\beta$, *alpha* values represent the coefficients of the AR model and MA model respectively. $\varepsilon _t$ expresses the randomness, c is the constant factor, $\theta _i$ indicates the numeric coefficient for the value associated with the *i*th lag, and $\varepsilon$ represents the residual. The differenced time series of ARIMA model is indicated by ${y_t}'= y_t-y_{t-1}$.

3 $$\begin{aligned} y_t= & {} \beta _0 + \beta _1 y_{t-1} + \beta _2 y_{t-2} +\cdots + \beta _p y_{t-p} +\varepsilon _t \end{aligned}$$

4 $$\begin{aligned} y_t= & {} c + \varepsilon _t + \theta _1 \varepsilon _{t-1} + \theta _2 \varepsilon _{t-2} +\cdots + \theta _q \varepsilon _{t-q} \end{aligned}$$

5 $$\begin{aligned} y_t= & {} \beta _1 y_{t-1} + \alpha _1 \varepsilon _{t-1} + \beta _2 y_{t-2} + \alpha _2 \varepsilon _{t-2} +\cdots + \beta _k y_{t-k}+ \alpha _k \varepsilon _{t-k} \end{aligned}$$

6 $$\begin{aligned} {y_t}'= & {} c + \beta _1 y'_{t-1} + \beta _2 y'_{t-2} + \alpha _2 \varepsilon _{t-2} +\cdots + \beta _p y'_{t-p} + \alpha _1 \varepsilon _{t-1}+\cdots + \alpha _q \varepsilon _{t-q} + \varepsilon _t \end{aligned}$$

The Augmented Dickey–Fuller test^34^ is used to check the stationarity of the time series. If the time series is stationary, we proceeded to AR, MA, or ARMA models and selected the best model among them using the lowest AIC (Akaike Information Criterion) and BIC (Bayesian Information Criterion) values^35^. Otherwise we had to take the difference to convert to stationary series and consider the ARIMA model with the rest of the models. Partial Autocorrelation (PACF) plots and Autocorrelation (ACF) plots^36^ were used to find the order of AR and MA respectively. The differencing order of the ARIMA model is based on the number of times we took the difference to make the series stationary. After identifying the model we simulate 10 time series from the selected model.

**Figure 3:**
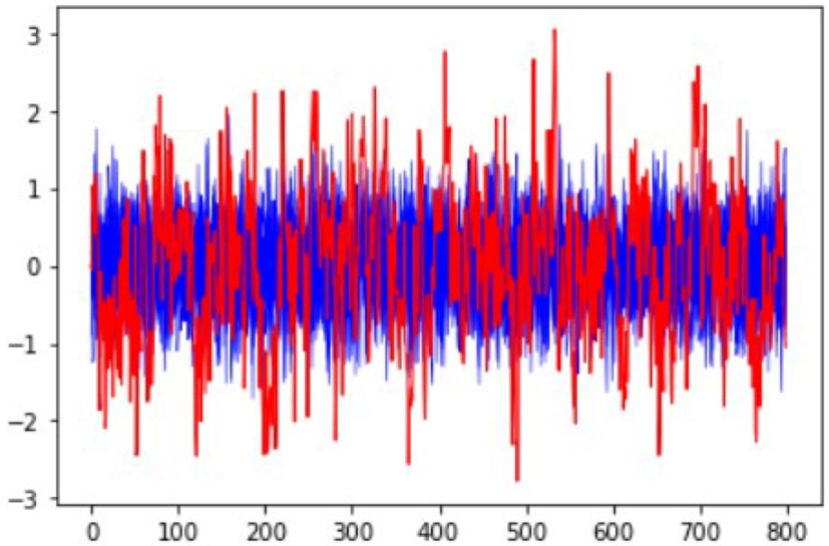
Simulated time series using state space model; blue indicates normal time series and red indicates abnormal time series.

The next step was generating an abnormal time series which showed unusual behavior compared to the rest of the time series. For that, we applied the AR(1) plus noise state-space model^37^ to generate our abnormal time series and it gave a distinguishable time series as in Fig. 3. The AR(1) plus state space model can be explained as follows.

7 $$\begin{aligned} z_t= & {} x_t + \varepsilon _t \end{aligned}$$

8 $$\begin{aligned} x_t= & {} \phi x_{t-1} + c + w_t \end{aligned}$$

Here Eq. (7) is the observation equation, and Eq. (8) is the state equation. $x_t$ denotes the state at time t, the transition matrix is $[\phi ]$, the observation matrix is [1], and the transition offset is *c*. The observation and transition noises, $WN(0,\sigma ^2)$ are indicated by $\varepsilon _t$ and $w_t$ respectively. The expectation of state is $E[X_t] = \frac{c}{1-\phi }$.

Now we have 11 time series: the first 10 time series are normal and the 11th series is abnormal. Using these 11 time series, we generated a median time series and calculated anomaly count, vertical distance, and DTW distance. Then we used the grid search optimization method^38^ to optimize the weights so that the abnormal sensor has the highest score. Also, the difference between the lowest score and the maximum score should be maximized. Here the score shows the abnormality of the sensor out of 100. The following algorithm 2 explains the weight-optimizing process.

**Algorithm 2:**
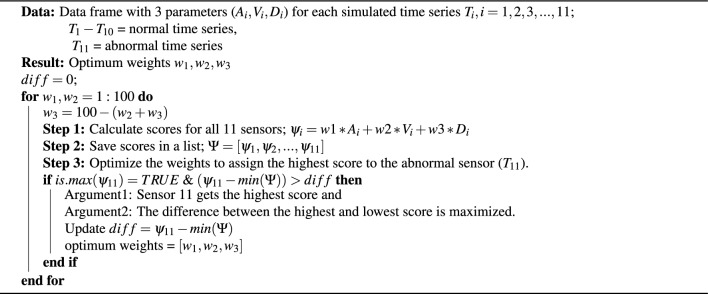
Grid search optimization to optimize weights

### Data collection and preparation

In order to test our algorithm, we used data from a school building in the USA and synthetic data which contains both collective and contextual anomalies. The school building is located in New York, USA. New York school building’s data^39^ were collected in 2018 from March to July (summer season), and 11 sensors were placed inside the building to collect data. Sensors were placed in different locations throughout the school building in a variety of different rooms, and collected temperature data over a period of time.

**Figure 4:**
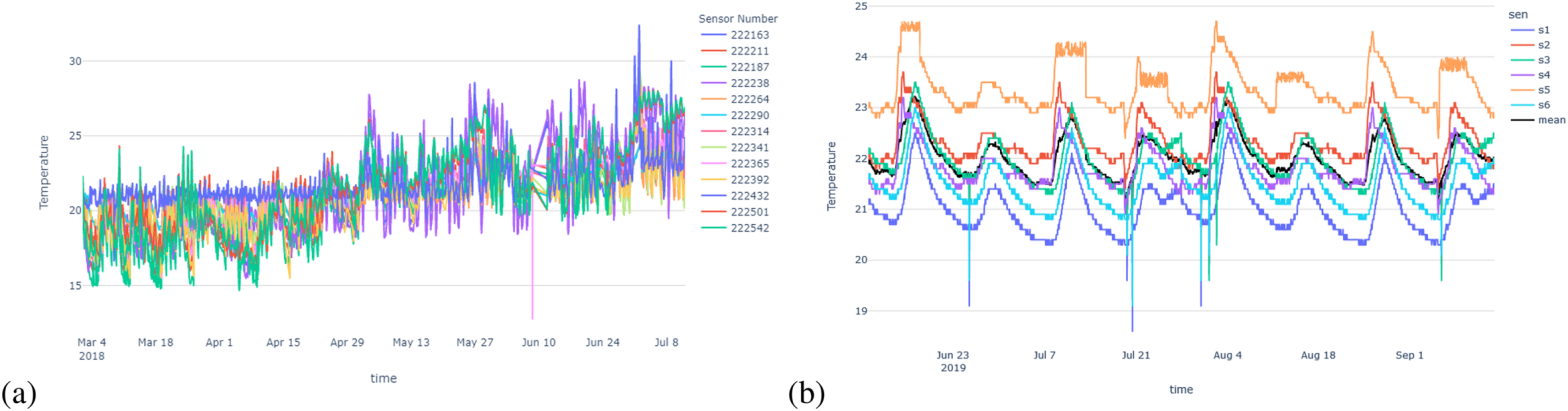
Temperature variation with time plots: (**a**) New York school building, (**b**) synthetic data.

For the synthetic dataset, we used an existing time series dataset and recreated similar time series with contextual anomalies and one different time series with collective anomalies. This synthetic dataset will help us to evaluate whether our method is capable of detecting the most abnormal time series due to collective anomalies. The following two plots in Fig. 4 show the variations and trends of the temperature time series of the synthetic data and the school building data.

Based on Fig. 4, in synthetic data, we can clearly see some collective outliers in the ‘orange’ color time series. Other time series are similar to mean time series, but they show some contextual (single) outliers. Since our algorithm should identify anomalies dues to environmental issues, it should identify the ‘orange’ color time series as the most abnormal one. In New York building’s we can see lots of abnormal points and sensor data with different patterns and higher variation. However, our method should be able to identify abnormal sensors in both scenarios, where the variation is high and low.

## Results

### Current anomaly detection methods vs DTW based anomaly detection method

**Figure 5:**
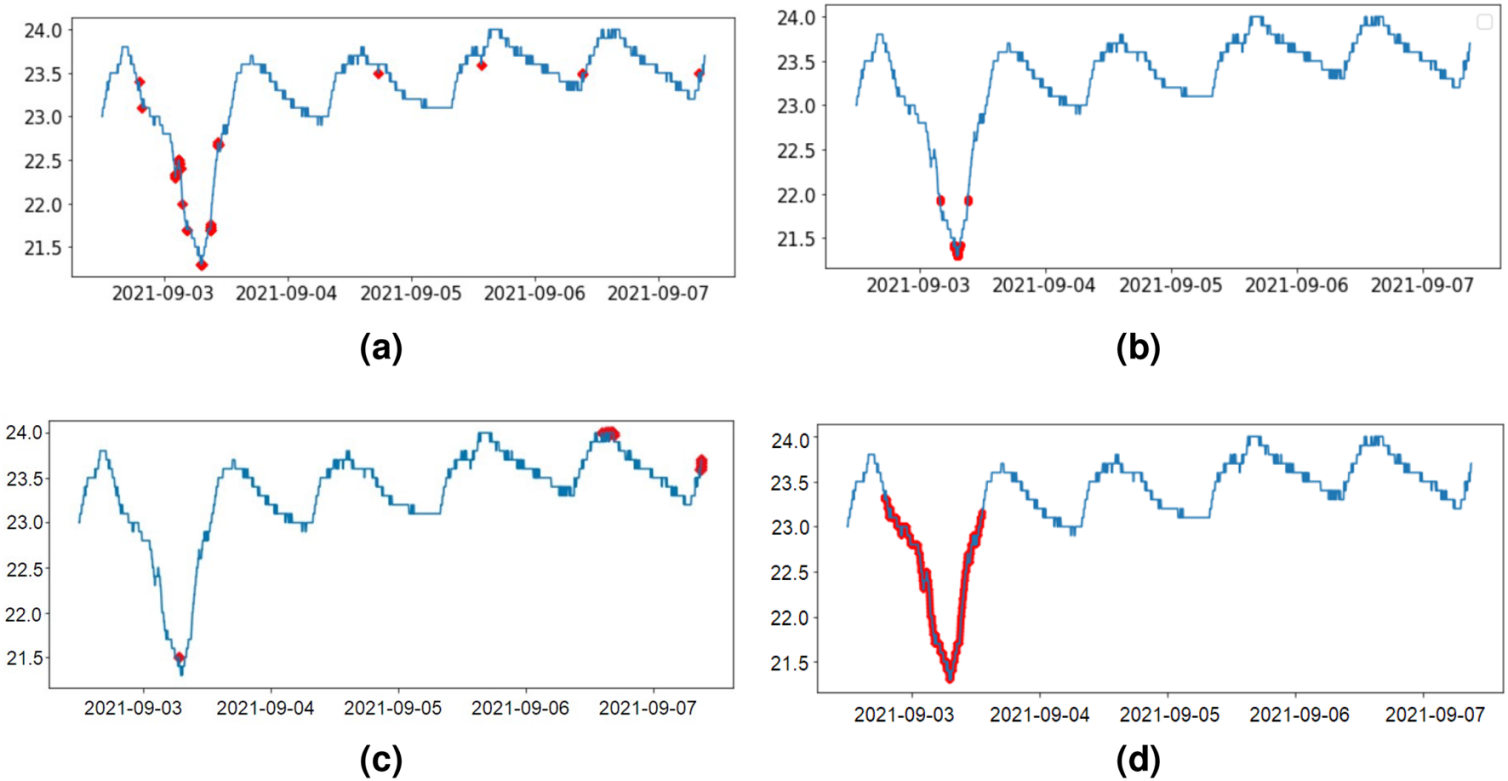
Results of anomaly detection methods: (**a**) STL decomposition method, (**b**) isolation forest method, (**c**) forecasting method, (**d**) DTW-based method. Anomaly points are shown in red color points.

Popular anomaly detection methods mainly identify abnormal points, not an abnormal time window. In this study, we tested the anomaly detection ability of some of these popular methods: STL decomposition, Isolation Forest, Forecasting method, and DTW method. Plots in Fig. 5 show a sample temperature time series from a building and how each different method identifies anomalies of that time series. Temperature sharply decreases from 23 to $21\,^{\circ }$C and increases back to $23\,^{\circ }$C within a short period of time. Facility managers would like to identify these type of time windows, which shows indoor environmental abnormal behaviors.

Based on the results in Fig. 5 we could see that only the DTW-based method identifies collective anomalies in a perfect way that we want to detect building anomalies. Hence we proceeded with the DTW-based method described previously. This method helped to identify abnormal time windows, not only the abnormal points, providing more applicable results in order to identify abnormal time series in buildings.

### Abnormal sensors detection

#### New York school building

**Figure 6:**
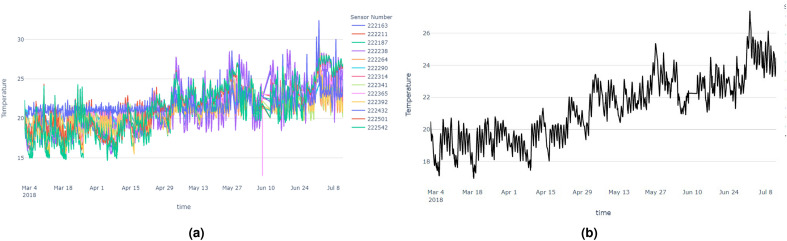
Time series plots of New York building (**a**) containing all the sensor’s temperature data, (**b**) median temperature time series.

In this section, we will discuss the results of the abnormal sensors detection process of the New York building. All the sensors are zone temperature thermostats, measuring temperature in a variety of room types. Room categories include gymnasiums, science wings, locker rooms, auditoriums, and more. As we explained in the methodology section, first we generated the median time series and considered it as the control time series. Figure 6 shows temperature vs time plots which contain all the sensors and the median time series of those sensor data.

**Figure 7:**
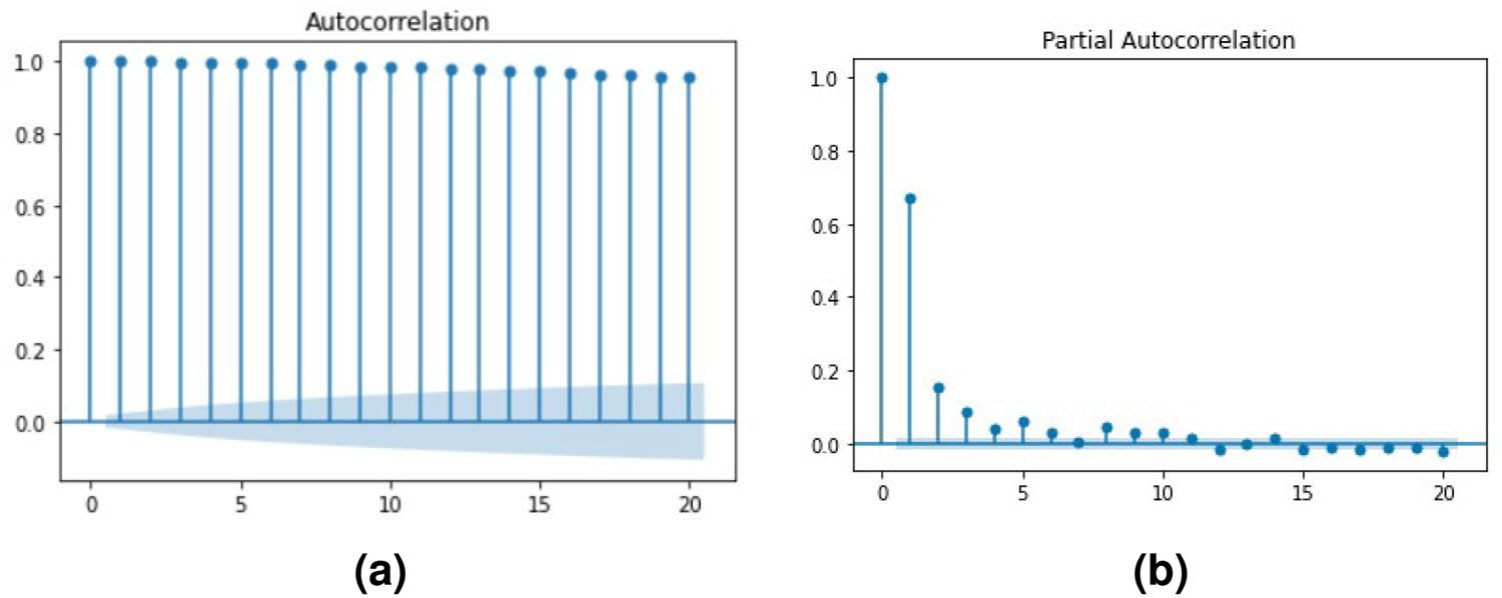
(**a**) Auto-correlation function (ACF) plot, (**b**) partial auto-correlation function (PACF) plot.

Then we needed to identify the time series model which fits best for the median time series. First, we checked the stationarity of the time series using the Augmented Dickey–Fuller test. Based on the results the time series was a non-stationary time series (p = 0.0835; failed to reject the null hypothesis). Then we had to choose the ARIMA model instead of the AR, MA, and ARMA models. After the 1st order differencing, the p-value drops beyond the 0.05 threshold. Hence we could consider the order of differencing as 1. To fit the models, we had to identify the order of AR and MA models and the following PCAF and ACF plots in Fig. 7 helped us to identify those orders.

Based on the PACF, since there are strong correlations till lag = 1, we assigned 2 as the order of the AR model. In the ACF plot, there are lots of strong correlations and because of that, we used 1 as the order of the MA model, as it is better to consider a less complex model. Using the orders of AR, and MA processes and order of differencing we fitted the ARIMA model, ARIMA(1,1,1). Then we used the estimated parameters of the ARIMA model to simulate the time series. The fitted ARIMA model is:

9 $$\begin{aligned} y'_t = 21.3274 + 0.8239* y'_{t-1} - 0.2899 * \varepsilon _{t-1} \end{aligned}$$

**Figure 8:**
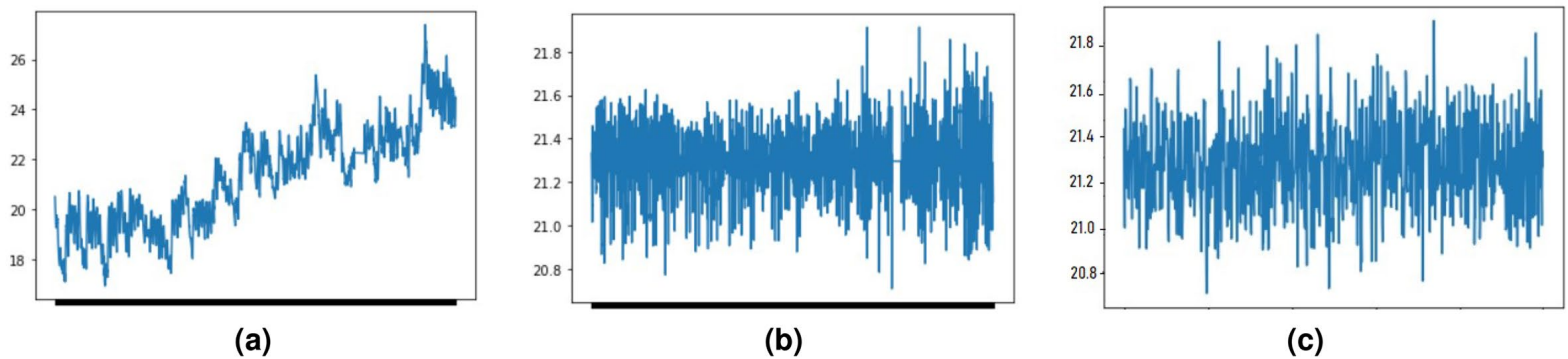
Plot (**a**) original time series, (**b**) de-trended time series, (**c**) simulated time series.

The original median time series had an increasing trend and using the differencing method we could detrend and make it stationary to fit the time series model. Once we detrended the time series we could compare the de-trended time series with the simulated time series. Figure 8 shows the original median, de-trended, and a simulated time series.

After that, we generated an abnormal time series and using the grid search, we explained in algorithm 2, we could optimize the weights of the score function ($\psi$). The final score function of *i*th time series of this building is as follows:

10 $$\begin{aligned} \psi _i = (70*A_i) + (29*V_i)+(1*D_i) \end{aligned}$$

**Table 2:** Result table of New York Building: the most abnormal sensor is highlighted in bold, the least abnormal sensor is highlighted in italics..

Sensor	Count	V_dist	DTW	Score	Rank
222163	0.6616	0.7501	0.5073	68.57	11
222211	0.1514	0.4352	0.3214	23.54	3
222187	0.3736	0.8155	0.6288	50.43	9
**222238**	**1.0000**	**1.0000**	**1.0000**	**100.00**	**13**
222264	0.4758	0.1464	0.3763	37.93	8
222290	0.3626	0.0000	0.1644	25.55	4
222314	0.0832	0.0387	0.0798	7.03	2
222341	0.1963	0.4584	0.2526	27.28	5
*222365*	*0.0000*	*0.1308*	*0.000*	*3.79*	*1*
222392	0.3423	0.2622	0.3287	31.89	7
222432	0.2168	0.5017	0.4455	30.17	6
222501	0.5323	0.7906	0.57.58	60.77	10
222542	0.7143	0.8163	0.7007	74.37	12

**Figure 9:**
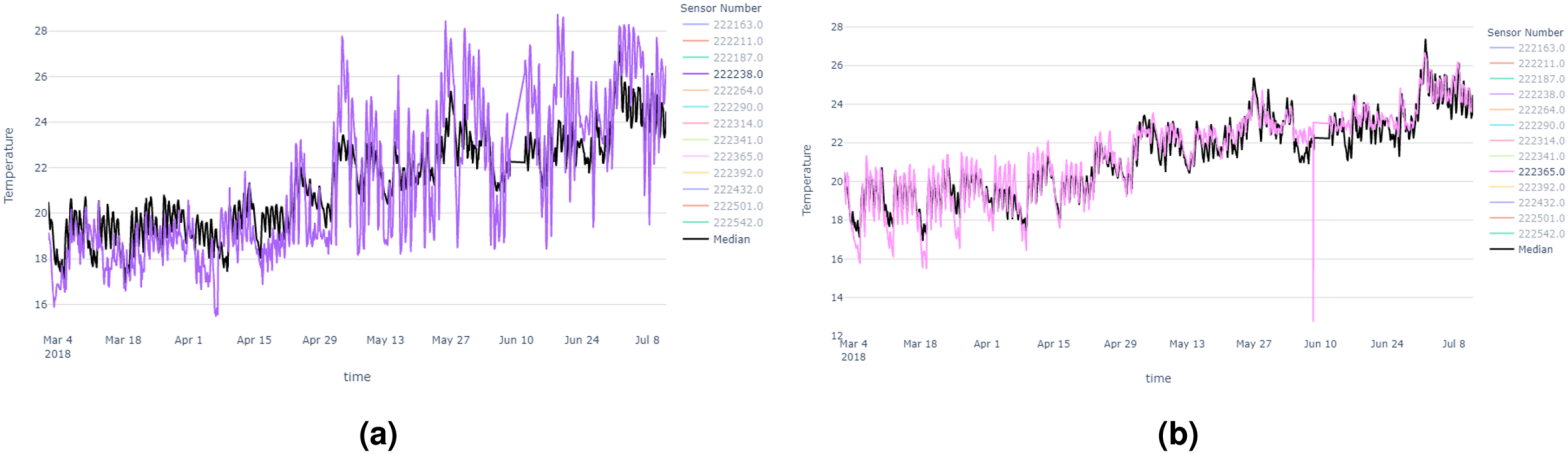
Time series plots of New York building: (**a**) The most abnormal sensor. (**b**) The least abnormal sensor.

The final score values and ranks of each sensor, based on the anomaly count, vertical distance and DTW distance compared to the real median time series, are shown in the following Table 2. Based on the table ‘sensor 222238’ can be considered as the most abnormal sensor while ‘sensor 222365’ is the least abnormal sensor. It can be clearly seen that ‘sensor 222365’ follows the median time series though it has one outlier point in the middle of the time series. Figure 9 shows the most abnormal and least abnormal sensors compared to the median time series.

#### Synthetic data set

**Figure 10:**
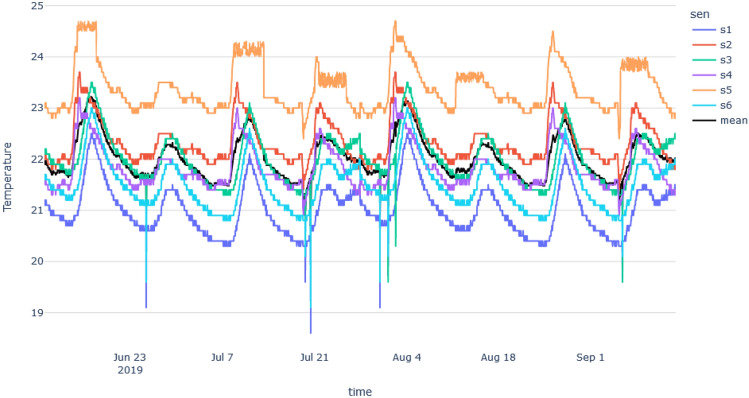
Time series of sensors in the synthetic data set.

This section explains the results of anomaly detection of synthetic data. As with the New York analysis, first, we had to take the median time series by considering the time series of all sensors. The time series of each sensor and median time series are shown in Fig. 10.

We checked the stationarity of the median time series which was found to be stationary (p-value = 0). The difference was taken in order to make the median time series stationary. Then we end up with ARMA (2,1) model as the best-fitted model. The fitted ARMA model is,

$$\begin{aligned} y_t =21.9194 - 0.7695* y_{t-1} - 0.2261* y_{t-2} + 0.1847 * \varepsilon _{t-1} \end{aligned}$$

Then we generated an abnormal time series and 10-time series using the selected model. Using those time series and grid search methods we found the weights for the score function of each *i*th time series as follows,

$$\begin{aligned} \psi _i = (31*A_i) + (68*V_i)+(1*D_i) \end{aligned}$$

After that, we calculated the actual anomaly count, mean vertical distance from an anomaly to the respective median time series points, and DTW distance for each sensor. Scores were found for each sensor using the score function which is summarized in the Table 3.

**Table 3:** Results table of Manitoba school building: the most abnormal sensor is highlighted in bold, the least abnormal sensor is highlighted in italics..

Sensor	Count	v_dist	DTW	Score	Rank
S1	0.594	0.594	0.593	59.44	4
S2	0.0000	0.0000	0.137	0.137	3
**3**	**0.00**	**0.00**	**0.00**	**0.00**	**1**
S4	0.00	0.00	0.046	0.046	2
*S5*	*1.0000*	*0.949*	*1.00*	*96.591*	6
S6	0.0000	1.0000	0.1406	68.25	5

**Figure 11:**
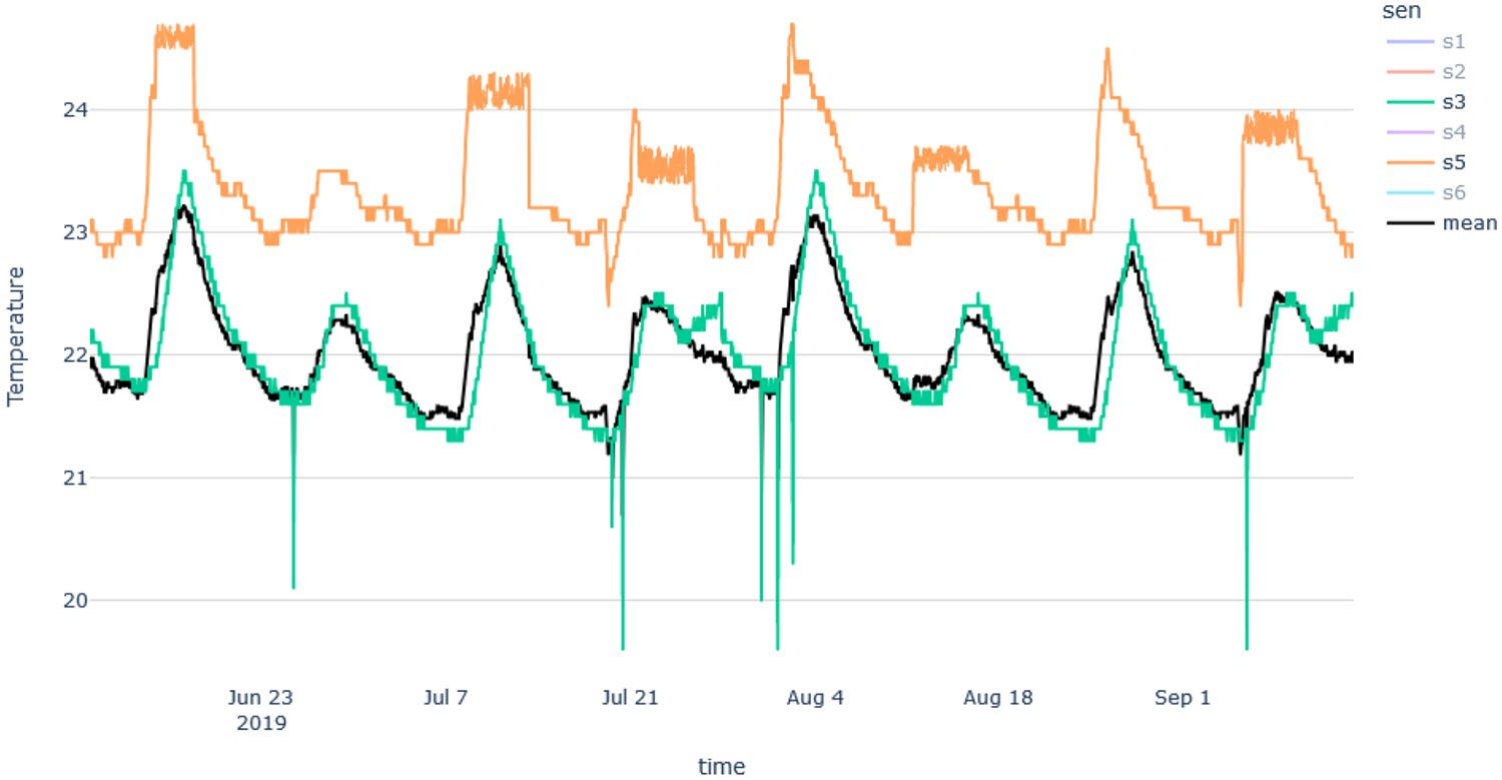
The most abnormal time series (orange), and the least abnormal time series (green), based on the median time series (black).

Based on the scores, the ‘sensor S5’ can be considered as the most abnormal sensor while the ‘sensor s3’ is the least abnormal sensor. Figure 11 shows the difference between these two time series plots. The most abnormal sensor clearly stands out from the median time series.

## Discussion and conclusion

This study was conducted in order to identify the locations with abnormal behaviors of indoor temperature, and we selected a school building and a synthetic dataset to test our method. Based on the results of those datasets, it is clearly shown that our algorithm using the weighted scoring system is capable of identifying sensors with abnormal behaviors. Sensors with low scores show a better alignment with the median time series while the sensors with higher scores show significant changes in the pattern of time series when compared with the median series.

Identifying abnormal performances of buildings is important for many parties in different ways. It helps building occupants improve their own comfort, while owners can save money by reducing energy waste and utility costs. Hence anomaly detection in buildings is an interesting research work, which is attracted by many building owners, tenants and researchers.

Our study offers significant advantages over existing models: it can effectively identify anomalies without the need for labeled training data, making it highly versatile even with a small dataset. Furthermore, this method does not depend on the distribution of time series data, enhancing its applicability. Despite its simplicity, it effectively identifies anomalies based on domain knowledge in the field of building science.

This anomaly detection process in buildings can help identify areas of concern that are difficult to find observationally or require a significant and costly energy audit to diagnose. These anomalies can be tied back to issues with a building’s mechanical system, spaces that are not conditioned to their real occupancy use, inefficient control systems, inefficient building envelope, insulation or window issues, and beyond. Anomaly detection could also help identify thermostats or other data collection equipment that are poorly performing or miscalibrated and may be transmitting false data that has a negative impact on that space.

However, an additional benefit of this methodology is to be able to test a building on multiple occasions to verify long-term trends and any degradation or loss of indoor environmental quality over time. A pattern of increasing frequency or severity in anomaly detection can be a key indicator of failing or degrading building conditions. Preventative analytics and fault detection can help to identify the presence of these issues before they reach a failure point, which will allow facilities managers to proactively identify and resolve known issues.

Though we used the median time series as the control time series, building owners and operators can change it to a known control time series, if available within the building. For example, if the building is on a temperature setpoint schedule, instead of the median time series, they can easily change this model to identify sensors that do not follow the setpoint schedule.

Not all anomalies are ignorable, and sometimes we do not want to consider some series as abnormal series, though we have some degree of abnormality. Hence, for future work, we are planning to improve our algorithm in a way that can identify whether the anomaly is acceptable or ignorable automatically. In this study, we only considered univariate anomalies, but in the future, we would like to develop a multivariate abnormal sensor detection algorithm as it can help to identify the performance issues of the building, quicker and more accurately than a human could.

## Data Availability

Data used in this paper is available upon request. Please contact the corresponding author (A.W.) for data.
